# Facet joint parameters which may act as risk factors for chronic low back pain

**DOI:** 10.1186/s13018-020-01706-6

**Published:** 2020-05-24

**Authors:** Ming Yang, Naiguo Wang, Xiaoxin Xu, Yu Zhang, Gang Xu, Yvang Chang, Zhonghai Li

**Affiliations:** 1 Department of Orthopaedics, First Affiliated Hospital of Dalian Medical University, No. 5, Longbin Road, Dalian, 116600 People’s Republic of China; 2 Key Laboratory of Molecular Mechanism for Repair and Remodeling of Orthopaedic Diseases, Liaoning Province, People’s Republic of China; 3 Department of Spinal Surgery, Shandong Provincial Hospital Affiliated to Shandong University, Jinan, People’s Republic of China

**Keywords:** low back pain (LBP), risk factor, lumbar facet joint, facet orientation (FO), facet tropism (FT), osteoarthritis (OA)

## Abstract

**Background:**

Facet orientation (FO) and facet tropism (FT) are two important structural parameters of lumbar facet joint. The purpose of this study was to evaluate the association between facet joint parameters and chronic low back pain (LBP).

**Methods:**

From June 2017 to January 2019, a total of 542 cases were enrolled in this study. There were 237 males and 305 females with a mean age of 35.8 years (range 18~59 years). All the cases were divided into a LBP group (LBP group) and a non-LBP group (N-LBP group) in this study. We compared their clinical parameters and facet joint parameters between two groups.

**Results:**

The LBP group was composed of 190 male and 252 female, whose ages ranged from 17 to 59 years (35.6 ±7.9 y). The N- LBP group was composed of 47 male and 53 female, whose ages ranged from 18 to 59 years (35.9 ± 7.5 y). Of these parameters, BMI (*P* = 0.008) and FT (*P* = 0.003) at all three levels were found to be significantly associated with incidence of chronic LBP (*P* < 0.05), but FO were only found to be significant at L3-L4 level and L5-S1 level (*P* < 0.05). Logistic regression analysis showed that high BMI and large FT were significant risk factors for chronic LBP (*P* < 0.05), and FT were found to might be independent risk factors for chronic LBP.

**Conclusion:**

FT may play a more important role in the pathogenesis of chronic LBP.

## Background

Low back pain (LBP) is the second most common complaint encountered by primary care physicians. It is associated with more disability than any other condition [1–4]. LBP is a presenting symptom for a myriad of disorders, some of which do not involve the spinal column. For those patients with back pain originating in the spine, an exact pathologic diagnosis is many times elusive. The inability to identify the exact cause of LBP in many patients leads to difficulty in treating the condition.

When LBP lasts for less than a month, it is said to be acute, for between 1 and 3 months subacute, and beyond that, chronic. Chronic LBP, with an estimated annual prevalence of 15~45% and a lifetime prevalence of 23%, is associated with significant medical and socioeconomic problems [5–7]. Specific causes of LBP are uncommon, and in approximately 90% of patients a specific generator cannot be identified with certainty [8, 9]. Clinical examination is not accurate in diagnosing the source of the chronic LBP. Potential sources of chronic LBP of the spinal column include the facet joints, sacroiliac joints, and intervertebral disks. These sources of pain were classified as non-specific LBP [1, 10–12]. They differ from secondary or specific back pain, which has a number of different causes, and may be spinal or extra-spinal (infection, inflammation, tumor, trauma).

Facet joints are complex three-dimensional structures, which serve in a multiplanar biomechanical capacity as osseous stabilizers of the posterior spinal column. They play an important role in maintaining stability of the lumbar spine by sharing load in compression and extension, and protecting the disc from excessive shear and rotational forces. Facet orientation (FO) and facet tropism (FT) are two important structural parameters of lumbar facet joint [13–15]. FO and FT may be associated with degenerative changes in the facet joints, either as the cause of degenerative changes or as the result of abnormal forces produced by degeneration. FO is the angle of the facet joint in the transverse view relative to the coronal plane. FT is defined as asymmetry between the left and right facet joint angles, with one joint having a more sagittal orientation than the other [16, 17]. Many previous studies have focused on the relationship between facet joint parameters and lumbar disc herniation or degenerative spondylolisthesis [15, 16, 18–21]. Nevertheless, the results of these studies were inconclusive [22–24]. Additionally, data on studies investigated the effects of FO and FT on chronic LBP appear only rarely in the literature. Therefore, the aim of this study was to evaluate the association between FO and FT, and chronic LBP in an Chinese population sample.

## Methods

### Patient Population

All the cases in this study undergoing multidetector CT scan were asked to complete the modified Nordic Low Back Questionnaire [25]. The first question on this questionnaire was: “Have you had low back pain on most days of at least 1 month in the last 12 months?” Individuals, who answered “yes,” or “no” on the above question, were categorized in the present study as the LBP outcome (dichotomous index). Similar methods are widely used in studies of work related LBP [12, 26, 27].

These patients had been referred to our hospital for diagnostic evaluation and treatment of bilateral chronic LBP, and had undergone CT scanning of their lumbar spine. In addition, 108 patients with digestive system diseases who underwent abdominal CT examination and hadn’t chronic LBP, were also included as a control group in this study. Patients with malformation (2 patients), previous spinal surgery or trauma (5 patients), inflammatory disease, spondylolisthesis (7 patients), myopathy, degenerative lumbar scoliosis (4 patients) or intervertebral disc herniation (9 patients) or radiculopathy, were excluded from this study. All the patients or relatives gave informed consent to participate in this study. Finally, a total of 542 cases were enrolled in this study. There were 237 males and 305 females with a mean age of 35.8 years (range 18~59 years).

### Data collection and outcome evaluations

**Fig. 1:**
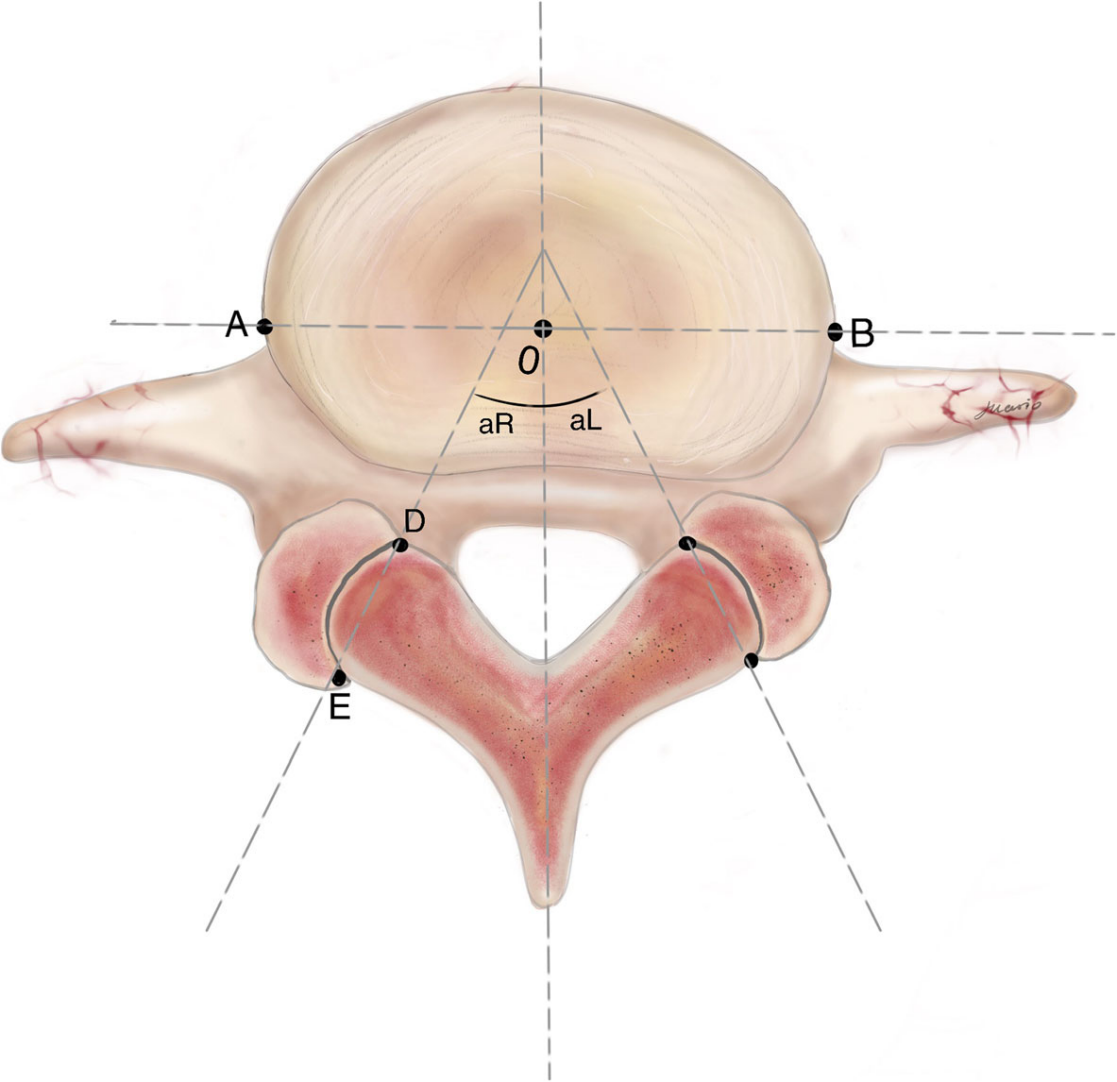
Diagram of the method used to measure the facet joint angle. The facet line is drawn between the 2 peaks of each of the superior articular facets (D and E). The midline is drawn through the center of the lumbar vertebral body (O, AO = OB) and the middle point of the base of the spinous process. The angle between the midsagittal line and facet line was measured for each side of the lumbar vertebral body (αR = right facet angle, αL = left facet angle). Facet orientation = (αR+αL)/2; Facet tropism = |αR-αL|

All the cases were divided into a LBP group (LBP group) and a non-LBP group (N-LBP group) in this study. We compared their clinical parameters [age, gender, body mass index (BMI), diabetes mellitus, current smoking, sports activity, occupational lifting, occupational driving], and facet joint parameters (FO and FT). Facet joint parameters were measured on the axial CT images at L3-L4, L4-L5, and L5-S1, using bone windows by using the method described by Noren et al and Li et al (Fig. 1) [17, 21].

A reading protocol for evaluation of FO and FT based on the above measuring method was developed. Using this protocol, one experienced musculoskeletal radiologist and two experienced spine surgeons analyzed the selected axial images. They were asked to do the measurements independently without referring back to previous imagines where they had completed the analyses. Measurements were repeated after 2 weeks with the same protocol. Inter- and intra-observer repeatability were calculated using an intra-class correlation coefficient, ICC (1, 3), formula [28]. The intra-observer reliability for grading different FO and FT indexes varied between 0.73 and 0.95. The inter-observer reliability ranged from 0.67 to 0.93. This range of kappa statistics represents fair to excellent reproducibility.

### Statistical analysis

The Statistical Package for Social Sciences software for Windows (Ver. 17.0, SPSS Inc, Chicago, IL) was used for the analysis. Age conformed to the normal distribution, and it was expressed as the mean ± standard deviation. The other measurement data did not conform to the normal distribution, so these parameters were expressed as the median (minimum, maximum). Univariate analysis was performed using One-Way ANOVA, Pearson χ2 test and Mann-Whitney U for potential parameters. Univariate and multiple logistic regression analysis was used to evaluate the effect of each factor on the chronic LBP. All of the above analysis and tests were with a *p* value of < 0.05 considered statistically significant.

## Results

The LBP group was composed of 190 male and 252 female, whose ages ranged from 18 to 59 years (35.6 ±7.9 y). The N- LBP group was composed of 47 male and 53 female, whose ages ranged from 18 to 59 years (35.9 ± 7.5 y). The results of univariate analysis of parameters were summarized in Table 1. Of these parameters, BMI (*P* = 0.008) and FT (*P* = 0.003) at all three levels were found to be significantly associated with incidence of chronic LBP (*P* < 0.05), but FO were only found to be significant at L3-L4 level and L5-S1 level (*P* < 0.05). Univariate and multiple logistic regression analysis was used to evaluate the effect of each factor on the chronic LBP (Table 2). Logistic regression analysis showed that high BMI and large FT were significant risk factors for chronic LBP (*P* < 0.05), and FT were found to might be independent risk factors for chronic LBP.

**Table 1:** FO and FT for chronic LBP at three levels using univariate analysis

Variable	Non-LBP Group (*n* = 100)	LBP Group (n =442)	*P*
Age (ys)	35.88±7.45	35.55±7.89	0.421
Gender (male:female)	47:53	190:252	0.465
BMI	22.63(17.11~32.69)	23.43(17.11~33.59)	0.008
Current smoking	21(21.00%)	100(22.62%)	0.725
Alcohol	5(5.00%)	31(7.01%)	0.465
Diabetes mellitus	11(11.00%)	56(12.67%)	0.647
Occupational lifting	31(31.00%)	143(32.35%)	0.794
Occupational driving	33(33.00%)	144(32.58%)	0.935
Sports activity	29(29.00%)	128(28.96%)	0.994
L3-L4			
FO(°)	33.83(13.50~58.60)	36.83(4.60~60.30)	0.046
FT(°)	2.85(0.00~35.80)	4.45(0.00~26.00)	<.001
L4-L5			
FO(°)	43.90(16.50~65.70)	45.40(5.50~91.35)	0.229
FT(°)	2.70 (0.00~17.30)	4.70(0.00~47.10)	<001
L5-S1			
FO(°)	49.23(27.00~78.35)	52.50(18.20~86.60)	0.007
FT(°)	3.40(0.10~27.30)	5.60(0.00~53.40)	<.001

LBP: low back pain; BMI: body mass index; FO: facet orientation; FT: facet tropism

**Table 2:** FO and FT for chronic LBP using univariate and multiple logistic regression analysis

Variable	Univariate Logistic Regression Analysis			Multiple Logistic Regression Analysis		
	OR	95% CI	*P*	OR	95% CI	*P*
BMI	1.096	1.023~1.174	0.009	1.076	1.003~1.153	0.040
L3-L4						
FO	1.016	0.992~1.040	0.191			
FT	1.141	1.068~1.219	<.001	1.119	1.048~1.196	0.001
L4-L5						
FO	1.010	0.989~1.032	0.339			
FT	1.104	1.045~1.166	<.001	1.086	1.026~1.150	0.005
L5-S1						
FO	1.029	1.008~1.052	0.008	1.030	1.006~1.055	0.013
FT	1.067	1.020~1.116	0.005	1.051	1.005~1.099	0.029

LBP: low back pain; BMI: body mass index; FO: facet orientation; FT: facet tropism

## Discussion

The cause of chronic LBP in cases without clear and serious anatomic pathology is not known. The serious structural lesions such as tumors, infection, fractures, and severe deformities are frequently painful and fortunately can be diagnosed with modern imaging studies. However, these patients with serious structural problems are uncommon. Much more commonly people have back pain episodes of varying degrees and either do not seek care or are treated symptomatically without a pathoanatomic diagnosis. Why some people with common backache become patients with serious disability is of enormous clinical and public health importance. Previous studies suggested that structural factors, exposure to mechanical stressors, psychological factors and social circumstances could be correlate with the development of chronic LBP [1, 2, 4–6, 9, 29]. However, there are many debates regarding the risk factors of chronic LBP and it is very difficult to define them because many complicated parameters are involved [29–32]. Therefore, an understanding of the relationship between pathoanatomic abnormality and advanced degeneration is of importance from a clinical and public health perspective.

Many researchers believed that LBP, as a result of lumbar degeneration, begins in the intervertebral disc, followed by spine malalignment and facet joint degeneration [33–35]. It is quite likely that the intervertebral disc and facet joints contribute to the initial degenerative process. In this study, we analyzed the associations between facet joint parameters (FO and FT), and chronic LBP in a Chinese population sample. To our knowledge, this work represents the first study to date to evaluate the associations between facet joint parameters and chronic LBP.

The facet joints are the only synovial joints in the spine, with hyaline cartilage overlying subchondral bone, a synovial membrane and a joint capsule. The intervertebral disc and the facet joints form a three-joint complex. As an important part of the three-joint complex in the posterior area of the spinal column, the lumbar facet joint has a far-reaching influence on the spine. Many biomechanical studies have considered that the intervertebral disc and the two facet joints carry loads together in the normal lumbar spine [36–38]. Any abnormality of one joint could affect the others, which might cause asymmetric stress transmission to both facet joint and corresponding disc, and this leads to stress concentration at particular regions of disc and facet joint.

Biomechanically, the facet joints primarily share the load in compression, extension, and torsion of the lumbar spine and protect the disc against torsion. Previous studies have found that FO and FT significantly influence the biomechanics of the corresponding segment [24, 37, 39–41]. Some scholars proposed that a more sagittal orientation of the facet joint promoted anterior gliding by reducing resistance to anterior shear forces [37, 41]. Kim et al. [37] studied three models at different FOs (50^o^, 55 ^o^, and 60^o^ relative to the coronal plane) and one model with FT (50 ^o^ on the right, 60 ^o^ on the left). The three models with different FOs did not differ in the intradiscal pressure gradient but the FT model had the greatest increase in intradiscal pressure and facet contract force, suggesting that tropism is what makes it more vulnerable to anterior sheer force than orientation. In addition, when tropism was present, the motion segment was found to have a tendency to rotate towards the more oblique joint when axial loads were applied. This asymmetric axial rotation caused by tropism can place additional torsional loads on the intervertebral discs which can lead to intervertebral disc injury and degeneration.

Several studies have examined the relationship between FO, FT and facet joint osteoarthritis (OA) [12, 22, 42–44]. Grogan et al. [42] found an association between FT and facet sclerosis, although no association was found between FT and a composite score of cartilage degeneration and sclerosis. Conclusions from this study are limited due to the very small sample size (n = 22) and the somewhat arbitrary criteria used to grade degeneration. Liu et al. [44] studied asymmetric facet joint OA and its relationship to FT and ligamentum flavum thickening using CT scans of L3-4, L4-5, and L5-S1 levels of the patients. The investigators did conclude that there was a positive correlation between FT, asymmetric facet joint OA, and ligamentum flavum thickness. Fujiwara et al. [43] found a significant association between FO and facet joint OA, but a negligible association between FT and OA in 111 Japanese patients. In a population-based CT study, a significant association between sagittal orientation and OA of the lumbar facet joints at the L4–L5 spinal level was found in 188 individuals. However, no association was found between FT and facet joint OA at any spinal level [22].

We found a significant difference in FT between non-LBP and LBP groups in the current study. Does this indicate that FT plays an important role in the production of LBP? Data on studies investigated the effects of FO and FT on chronic LBP appear only rarely in the literature. For this reason,, we compared the FO and FT in depth between the non-LBP and LBP groups in an Chinese population sample, and found that there was a significant correlation between FT and chronic LBP. We considered that both sides of the facet joints and intervertebral disc together constitute the spinal three-joint complex. When the lumbar spine is flexed and twisted, if both joints are asymmetric, the stress of the three-joint complex is imbalanced. Resistance on the sides of the vertebral body is different, and the vertebral body will deviate from the original trajectory, thus pulling the rear of the intervertebral disc. Such a loading imbalance may accelerate the degeneration of the facet joints and intervertebral discs. Therefore, FT may play a more important role in the pathogenesis of chronic LBP.

### Limitations

Our study has several limitations. This was a cross-sectional observational study on facet angle. our study was limited by geometrical considerations. Even if facet joints often were not planar, our measurements did not take into account the complex three-dimensional geometry of the facet joints and their relationship with the disc and facet joint degeneration. Due to these limitations, future studies should focus on more sophisticated biomechanical factors of the lumbar spine, and further explore the correlation between biomechanical factors and chronic LBP. Accordingly, we plan to perform next the biomechanical analysis of facet configuration such as FT and FO in finite element models of lumbar spine.

## Conclusions

The current study showed that FT were found to be independent risk factors for chronic LBP. FT may play a more important role in the pathogenesis of chronic LBP. The exact mechanism between facet joint parameters and chronic LBP warrants further investigation.

## Data Availability

All data used and analyzed during this study are available from the corresponding author upon reasonable request.

## Abbreviations

FO: Facet orientation FT Facet tropism LBP Low back pain OA Osteoarthritis BMI Body mass index

## References

[1] Chenot JF, Greitemann B, Kladny B (2017). Non-Specific Low Back Pain. Deutsches Arzteblatt international.

[2] Delitto A, George SZ, Van Dillen L (2012). Low back pain. The Journal of orthopaedic and sports physical therapy.

[3] Casser HR, Seddigh S, Rauschmann M (2016). Acute Lumbar Back Pain. Deutsches Arzteblatt international.

[4] Rao D, Scuderi G, Scuderi C (2018). The Use of Imaging in Management of Patients with Low Back Pain. Journal of clinical imaging science.

[5] European Commission CBMC (2002). European guidelines for the management of low back pain. Acta orthopaedica Scandinavica. Supplementum.

[6] Martin BI, Deyo RA, Mirza SK (2008). Expenditures and health status among adults with back and neck problems. JAMA : the journal of the American Medical Association.

[7] Katz Jeffrey N. (2006). Lumbar Disc Disorders and Low-Back Pain: Socioeconomic Factors and Consequences. The Journal of Bone and Joint Surgery (American).

[8] Finch P (2006). Technology Insight: imaging of low back pain. Nat Clin Pract Rheumatol.

[9] Ract I, Meadeb JM, Mercy G (2015). A review of the value of MRI signs in low back pain. Diagnostic and interventional imaging.

[10] Van Zundert J, Hartrick C, Patijn J (2011). Evidence-based interventional pain medicine according to clinical diagnoses. Pain practice : the official journal of World Institute of Pain.

[11] Bogduk N (1995). The anatomical basis for spinal pain syndromes. J Manip Physiol Ther.

[12] Kalichman L, Li L, Kim DH (2008). Facet joint osteoarthritis and low back pain in the community-based population. Spine (Phila Pa 1976).

[13] Alonso F, Kirkpatrick CM, Jeong W (2017). Lumbar Facet Tropism: A Comprehensive Review. World neurosurgery.

[14] Kalichman L, Hunter DJ (2007). Lumbar facet joint osteoarthritis: a review. Semin Arthritis Rheum.

[15] Schleich C, Muller-Lutz A, Blum K (2016). Facet tropism and facet joint orientation: risk factors for the development of early biochemical alterations of lumbar intervertebral discs. Osteoarthr Cartil.

[16] Lee DY, Ahn Y, Lee SH (2006). The influence of facet tropism on herniation of the lumbar disc in adolescents and adults. The Journal of bone and joint surgery British volume.

[17] Noren R, Trafimow J, Andersson GB (1991). The role of facet joint tropism and facet angle in disc degeneration. Spine (Phila Pa 1976).

[18] Gao T, Lai Q, Zhou S (2017). Correlation between facet tropism and lumbar degenerative disease: a retrospective analysis. BMC Musculoskelet Disord.

[19] Wang H, Zhou Y (2016). Facet tropism: possible role in the pathology of lumbar disc herniation in adolescents. J Neurosurg Pediatr.

[20] Chadha M, Sharma G, Arora SS (2013). Association of facet tropism with lumbar disc herniation. European spine journal : official publication of the European Spine Society, the European Spinal Deformity Society, and the European Section of the Cervical Spine Research Society.

[21] Li Z, Yang H, Liu M (2018). Clinical Characteristics and Risk Factors of Recurrent Lumbar Disk Herniation: A Retrospective Analysis of Three Hundred Twenty-One Cases. Spine (Phila Pa 1976).

[22] Kalichman L, Suri P, Guermazi A (2009). Facet orientation and tropism: associations with facet joint osteoarthritis and degeneratives. Spine (Phila Pa 1976).

[23] Vanharanta H, Floyd T, Ohnmeiss DD (1993). The relationship of facet tropism to degenerative disc disease. Spine (Phila Pa 1976).

[24] Kong MH, He W, Tsai YD (2009). Relationship of facet tropism with degeneration and stability of functional spinal unit. Yonsei Med J.

[25] Kuorinka I, Jonsson B, Kilbom A (1987). Standardised Nordic questionnaires for the analysis of musculoskeletal symptoms. Appl Ergon.

[26] Ghaffari M, Alipour A, Jensen I (2006). Low back pain among Iranian industrial workers. Occup Med.

[27] Dovrat E, Katz-Leurer M (2007). Cold exposure and low back pain in store workers in Israel. Am J Ind Med.

[28] Shrout PE, Fleiss JL (1979). Intraclass correlations: uses in assessing rater reliability. Psychol Bull.

[29] Hancock MJ, Maher CM, Petocz P (2015). Risk factors for a recurrence of low back pain. The spine journal : official journal of the North American Spine Society.

[30] Maatta JH, Wadge S, MacGregor A (2015). ISSLS Prize Winner: Vertebral Endplate (Modic) Change is an Independent Risk Factor for Episodes of Severe and Disabling Low Back Pain. Spine (Phila Pa 1976).

[31] Luoma K, Vehmas T, Kerttula L (2016). Chronic low back pain in relation to Modic changes, bony endplate lesions, and disc degeneration in a prospective MRI study. European spine journal : official publication of the European Spine Society, the European Spinal Deformity Society, and the European Section of the Cervical Spine Research Society.

[32] Suri P, Fry AL, Gellhorn AC (2015). Do Muscle Characteristics on Lumbar Spine Magnetic Resonance Imaging or Computed Tomography Predict Future Low Back Pain, Physical Function, or Performance? A Systematic Review. PM & R : the journal of injury, function, and rehabilitation.

[33] Butler D, Trafimow JH, Andersson GB (1990). Discs degenerate before facets. Spine (Phila Pa 1976).

[34] Kong MH, Morishita Y, He W (2009). Lumbar segmental mobility according to the grade of the disc, the facet joint, the muscle, and the ligament pathology by using kinetic magnetic resonance imaging. Spine (Phila Pa 1976).

[35] Fujiwara A, Tamai K, An HS (2000). The relationship between disc degeneration, facet joint osteoarthritis, and stability of the degenerative lumbar spine. J Spinal Disord.

[36] Byrne RM, Zhou Y, Zheng L (2018). Segmental variations in facet joint translations during in vivo lumbar extension. J Biomech.

[37] Kim HJ, Chun HJ, Lee HM (2013). The biomechanical influence of the facet joint orientation and the facet tropism in the lumbar spine. The spine journal : official journal of the North American Spine Society.

[38] Adams MA, Hutton WC (1983). The mechanical function of the lumbar apophyseal joints. Spine (Phila Pa 1976).

[39] Adams MA, Hutton WC (1980). The effect of posture on the role of the apophysial joints in resisting intervertebral compressive forces. The Journal of bone and joint surgery. British volume.

[40] Yang KH, King AI (1984). Mechanism of facet load transmission as a hypothesis for low-back pain. Spine (Phila Pa 1976).

[41] Toyone T, Ozawa T, Kamikawa K (2009). Facet joint orientation difference between cephalad and caudad portions: a possible cause of degenerative spondylolisthesis. Spine (Phila Pa 1976).

[42] Grogan J, Nowicki BH, Schmidt TA (1997). Lumbar facet joint tropism does not accelerate degeneration of the facet joints. AJNR Am J Neuroradiol.

[43] Fujiwara Atsushi, Tamai Kazuya, An Howard S., Lim Tae-Hong, Yoshida Hiroyuki, Kurihashi Akira, Saotome Koichi (2001). Orientation and Osteoarthritis of the Lumbar Facet Joint. Clinical Orthopaedics and Related Research.

[44] Liu HX, Shen Y, Shang P (2016). Asymmetric Facet Joint Osteoarthritis and Its Relationships to Facet Orientation, Facet Tropism, and Ligamentum Flavum Thickening. Clinical spine surgery.

